# A Comparative Study of the Electrical and Electromechanical Responses of Carbon Nanotube/Polypropylene Composites in Alternating and Direct Current

**DOI:** 10.3390/s22020484

**Published:** 2022-01-09

**Authors:** Abraham Balam, Raúl Pech-Pisté, Zarel Valdez-Nava, Fidel Gamboa, Alejandro Castillo-Atoche, Francis Avilés

**Affiliations:** 1 Centro de Investigación Científica de Yucatán A.C., Unidad de Materiales, Calle 43 No. 130 x 32 y 34, Col. Chuburná de Hidalgo, Mérida 97205, Yucatán, Mexico; abrahambalam.mena@gmail.com (A.B.); rpech.piste@gmail.com (R.P.-P.); 2 LAPLACE, Université de Toulouse, CNRS, INPT, UPS, 31062 Toulouse CEDEX 9, France; valdez@laplace.univ-tlse.fr; 3 Centro de Investigación y de Estudios Avanzados, Unidad Mérida, Departamento de Física Aplicada, Km. 6 Antigua Carretera a Progreso A.P. 73, Cordemex, Mérida 07360, Yucatán, Mexico; ffgamboa@cinvestav.mx; 4 Facultad de Ingeniería, Universidad Autónoma de Yucatán, Av. Industrias No Contaminantes por Periférico Norte A.P. 150, Cordemex, Mérida 97000, Yucatán, Mexico; acastill@correo.uady.mx

**Keywords:** carbon nanotubes, electrical properties, alternating current, electromechanical, piezoimpedance, piezoresistivity

## Abstract

The electrical and electromechanical responses of ~200 µm thick extruded nanocomposite films comprising of 4 wt.% and 5 wt.% multiwall carbon nanotubes mixed with polypropylene are investigated under an alternating current (AC) and compared to their direct current (DC) response. The AC electrical response to frequency (*f*) and strain (piezoimpedance) is characterized using two configurations, namely one that promotes resistive dominance (resistive configuration) and the other that promotes the permittivity/capacitive contribution (dielectric configuration). For the resistive configuration, the frequency response indicated a resistive–capacitive (RC) behavior (negative phase angle, *θ*), with a significant contribution of capacitance for frequencies of 10^4^ Hz and above, depending on the nanotube content. The piezoimpedance characterization in the resistive configuration yielded an increasing impedance modulus (|*Z*|) and an increasing (negative) value of *θ* as the strain increased. The piezoimpedance sensitivity at *f* = 10 kHz was ~30% higher than the corresponding DC piezoresistive sensitivity, yielding a sensitivity factor of 9.9 for |*Z*| and a higher sensitivity factor (~12.7) for *θ*. The dielectric configuration enhanced the permittivity contribution to impedance, but it was the least sensitive to strain.

## 1. Introduction

The addition of carbon nanotubes (CNTs) or other graphenic nanomaterials in sufficient concentrations to nonconductive polymers yields nanocomposites with the ability of electroconduction. The electrical resistance (*R*) of such nanocomposites is highly influenced by the filler volume fraction and the state of dispersion of the fillers, and it may also depend on the applied strain [1,2,3,4]. Many of the multifunctional and sensing capabilities of these CNT/polymer nanocomposites rely not only on electroconduction, but also on the dependence of the electrical conductivity on strain [5,6,7,8]. Among the sensing capabilities, the electrical response to strain, known as piezoresistivity for the case of direct current (DC), has been widely studied [3,5,9,10]. For DC, the literature reports higher piezoresistive sensitivity (gage factor) at lower CNT concentrations [3,11,12,13], linear response at low strains [14], and overall higher sensitivity for thermoplastic matrices than for thermosetting ones [3]. On other hand, the electromechanical response in alternating current (AC), known as piezoimpedance, has been considerably less studied [15,16,17,18,19,20,21]. The electrical impedance (*Z*) presents a more complete physical parameter to study than just the electrical resistance since it comprises not only the resistive contribution, but also the capacitive (*C*) and inductive (*L*) ones. In its polar representation, *Z* can be fully described by two parameters, namely the impedance modulus (|*Z*|) and its phase angle (*θ*). In this sense, it has been reported that polymeric composites based on carbon nanostructures can be considered as materials with a resistive–capacitive (*RC*) behavior, neglecting the contribution of *L* [15,22,23]. However, the contribution of each component (resistive and capacitive) to the total impedance of the material is frequently determined with the assumption of a (series/parallel) electrical circuit model [15,19,21,24,25], which is not unique. This causes such relative contributions to perhaps be debatable, since they depend on the chosen model. Thus, choosing a strain sensitivity parameter that depends on a nonunique model may be uncertain. For CNT/epoxy composites, it has also been observed that the piezoimpedance sensitivity depends on frequency (*f*), reporting higher sensitivities for higher *f* [15]. A few authors have used an approach, whereby fitting the impedance response to an electrical circuit model, a value is obtained for capacitance as a function of strain, thus yielding a “piezocapacitive” response [19,25]. In a novel study, Eddib and Chung proposed an impedance method which, according to the authors, allows extracting the contribution of the capacitance from the (measured) total impedance of the system [26]. A second work of the same research group [27], claims that this capacitive method is sensitive to damage (holes) generated on carbon fiber polymer-matrix composites. In such works, the piezocapacitance is not a direct measurement, but depends on certain assumptions, such as the assumption of a parallel RC model for data interpretation. As for the comparison between the piezoimpedance and piezoresistive responses, very few studies have been conducted to date. The few experimental data available on the topic (for CNT/epoxy) point to higher strain sensitivity for the AC response, which was ascribed to the concurrent action of the resistive and capacitive contributions [15,18]. The broadband (from DC to several MHz AC impedance response provides a more complete description of the material’s electrical response and is directly related to its composition and microstructure. In spite of this, research on the AC piezoimpedance response for thermoplastic nanocomposites, which are more deformable, is scarce. Systematic investigations comparing the electrical response to strain in DC to that in AC are very scarce, and the factors that affect the sensitivity to strain in AC are yet not fully understood.

Considering this motivation, the current work contributes a systematic study of the AC electromechanical (piezoimpedance) response of multiwall carbon nanotube/polypropylene (MWCNT/PP) composites. This is achieved by investigating the effect of the AC frequency on the strain sensing capabilities and comparing such a response to its DC counterpart. For comparison purposes, the piezoimpedance response is also studied using the method proposed by Eddib and Chung [26]. This method aims to extract the permittivity contribution of the material from the global electrical response of the material and measurement setup. Finally, the piezoresistive and piezoimpedance sensitivities (using two methods for piezoimpedance) are directly compared, investigating the role of the applied frequency. It is expected that the findings reported herein will advance the use of the AC electrical response for the self-sensing of strain and motion. This will assist further material developments on new flexible sensing devices for a myriad of applications, such as tactile sensing, motion monitoring, soft robotics, and human–machine interfaces.

## 2. Materials and Methods

### 2.1. Materials

Multiwall carbon nanotubes (MWCNTs) were acquired from Cheaptubes Inc., (Grafton, VT, USA). The MWCNTs were produced by chemical vapor deposition with an internal diameter of 4–10 nm, an external diameter of ~30 nm, and length of 1–6 μm. Nanotubes were dispersed in Formolene^®^ 1102KR polypropylene (PP) from Formosa Plastics Co., (Livingston, NJ, USA), with a melt index of 4 g/10 min.

### 2.2. Nancomposites Preparation

The MWCNT/PP nanocomposites were obtained by applying a two-step melt processing method. Initially, the MWCNTs were dispersed into PP at two weight concentrations (4 wt.% and 5 wt.%) using a batch mixer at 190 °C and 40 rpm for 10 min. Afterward, the MWCNT/PP blends were pulverized and fed to a three-zones single-screw Brabender extruder. All zones of the extruder were set to 190 °C, and the screw speed used was 30 rpm. A 12 cm wide slot die was fitted to the extruder with a die gap (lip opening) of 200 µm and a temperature of 190 °C. After extrusion, the films were automatically pulled using a Brabender take-off equipment, with ~200 µm gap between rollers and a linear speed of 0.4 m/min. This resulted in MWCNT/PP nanocomposite films with thicknesses ranging between 190 µm and 210 µm (with 200 µm as mean value). All test specimens were cut from the manufactured films along the extrusion (machine) direction.

### 2.3. Piezoresistive Characterization

To investigate the piezoresistive response of the MWCNT/PP composites, type III ASTM D638 standard specimens [28] (downscaled 3:1) were obtained from the meter-long films, with the longest (axial) dimension along the extrusion direction. The percolation threshold of these composites is close to 3 wt.% [13], so well percolated materials of 4 wt.% and 5 wt.% with a large signal to noise ratio were selected. Thus, five specimens of each MWCNT concentration (4 wt.% and 5 wt.%) were instrumented for electrical measurements by fixing two electrodes. Dedicated experiments (not shown) indicated negligible differences between four-wire and two-wire in situ electrical resistance measurements during strain application. The mean square error between the full piezoresistive curves obtained using two-wire and four-wire electrodes was only 2.1%. This negligible difference is because the initial (unloaded, *R*_0_) resistances were around 10^5^ Ω. Thus, the two-wire configuration was used herein for piezoresistive testing (DC).

**Figure 1:**
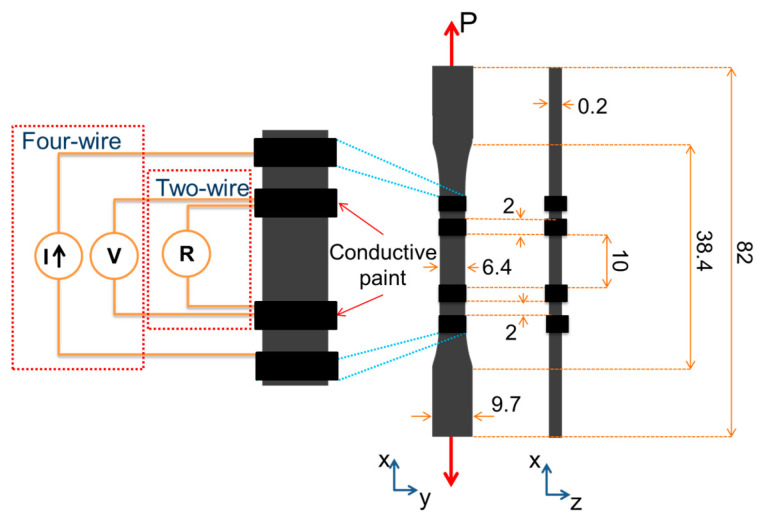
Piezoresistive and piezoimpedance (resistive configuration) specimen. Dimensions in mm.

To promote volumetric current flow, each electrode consisted of a 2 mm wide contour of conductive paint (Bare Conductive Ltd., London, UK) fixing AWG 38 copper wires. The electrodes were 10 mm apart, and the specimens were subjected to uniaxial loading (*P*) along the extrusion direction, as depicted in Figure 1. Mechanical loading was applied by a Shimadzu AGS-X universal testing machine with a 1 kN load cell, setting the crosshead speed to 1 mm/min. The load and crosshead stroke signals as well as the instantaneous electrical resistance of the specimen (*R*) were synchronized using a Keysight 34980A high-performance multifunction switch/measure unit with a 34921A 40-channel multiplexer module. The axial stress (*σ*) was calculated as *P* divided by the cross-sectional area. Since a film geometry does not allow the use of strain gages or an extensometer, the axial strain (*ε*) was obtained as the crosshead displacement divided by the specimen gage length (38.4 mm). As reported in [29], the selection of the distance between grips (38.4 mm in our case) as the calibrated gage length yielded adequate values of *ε* for specimens of these dimensions. Changes in electrical resistance (*ΔR = R − R*_0_) were calculated from the difference between the instantaneous *R* and its initial value before loading (*R*_0_). Then, the fractional change of electrical resistance (*ΔR/R*_0_) was used to define the sensitivity factor (*k_β_*) as

(1) $$k_{\beta }=\frac{∆\beta /\beta_{0}}{\varepsilon },$$

where *β* = *R* for the piezoresistive response.

Dedicated cyclic experiments (not shown) guaranteed mechanical reversibility (elastic behavior) for strains below *ε* = 1.2%. Since the electromechanical (piezoresistive) response exhibited a nonlinear behavior, the curves were split into two regions for analysis purposes, associating a sensitivity (gage) factor to each region. The first factor (*k_R_*_1_) was calculated within the elastic region (0 ≤ *ε* ≤ 0.8%), where the electromechanical response was deemed piezoresistive due to its reversibility. The second sensitivity factor (*k_R_*_2_) was calculated at the region 1% ≤ *ε* ≤ 3%. Higher strains (*ε* > 3%) were not considered in the quantification of sensitivity factors, since such high strain levels are associated with irreversible events and material failure.

### 2.4. Alternating Current Characterization

#### 2.4.1. Resistive Configuration

Alternating current (AC) characterization of 4 wt.% and 5 wt.% nanocomposites was carried out in two configurations. The first one, named “resistive configuration”, mimicked the piezoresistive configuration described in Figure 1. The second one was a different configuration recently proposed in the literature [26], named herein “dielectric configuration”. The dielectric configuration aims to extract the capacitive/permittivity contribution to the impedance. In the “resistive configuration” (Figure 1), impedance measurements were taken using the four-wire method. In this method, the impedance between the internal electrodes was determined from the relationship between the current (*I*) that circulates through the external electrodes and the electric potential drop (*V*) between the internal electrodes, see Figure 1. Measurements were performed using an LCR Keysight E4980A equipment, setting up the AC potential to 1 *V_rms_*. A 5-test replicate plan was conducted for all characterizations presented herein. First, the frequency response (impedance as a function of frequency) of MWCNT/PP composites was determined, measuring the impedance modulus (|*Z*|_0_) and phase angle (*θ*_0_) at zero strain (*ε* = 0). This was carried out for frequencies (*f*) up to 1 MHz. To estimate the relative contributions of the resistance (*R*) and capacitance (*C*) to the impedance of the nanocomposites, frequency response curves (both |*Z*|_0_ and *θ*_0_) were fitted to a parallel RC electrical circuit model, as is described in Section S.1 of the supplementary information. From the best fit parameters of the circuit model, equivalent *R* and *C* values were estimated for the two MWCNT concentrations.

Based on the results of the frequency response analysis, only *f* = 10 kHz and *f* = 100 kHz were considered for the piezoimpedance analysis. During uniaxial tensile loading until failure, instantaneous impedance modulus (*|Z|*) and phase angle (*θ*) were measured using the LCR equipment. The load (*P*) and crosshead displacement voltages were acquired by a Keysight 34980A high-performance multifunction switch/measure unit with a 34921A 40-channel multiplexer module. The measurements of the LCR and the multifunction equipment were synchronized by means of a proprietary data logger software based on LabView (NI, Austin, TX, USA). From the measured |*Z*| and *θ*, fractional changes of impedance modulus (*Δ|Z|/|Z|_0_*) and phase angle (*Δθ*/*θ*_0_) were determined. As for the piezoresistive response, piezoimpedance sensitivity factors were calculated for both impedance parameters by substituting *β* in Equation (1) for |*Z*| and *θ* and labeling them as *k_Z_* and *k_θ_*, respectively. Sensitivity factors were calculated for each impedance parameter and for identical strain levels as those used for the piezoresistive analysis (Section 2.3). This means *k_Z_*_1_ for |*Z*| and *k_θ_*_1_ for *θ* for *ε* ≤ 0.8%, and *k_Z_*_2_ and *k_θ_*_2_ for 1% ≤ *ε* ≤ 3.0%.

#### 2.4.2. Dielectric Configuration

**Figure 2:**
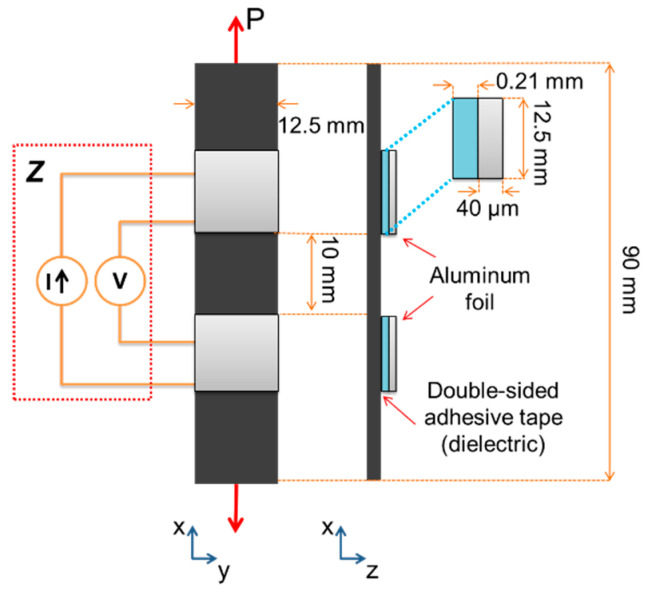
Specimen instrumented for piezoimpedance characterization in the dielectric configuration.

Recently, a method for measuring the permittivity of carbon fiber/polymer and carbon–carbon composites was proposed [26,27]. The method relies on fixing electrodes to the specimen in a way that increases the permittivity of the system. To this aim, the electrodes comprise a dielectric film placed between the specimen and the conductive electrode (aluminum foil). By assuming a parallel RC circuit model (resistance and capacitance in parallel), the total capacitance of the system is represented by an array of three capacitors in a series (electrode interface/specimen/electrode interface). Finally, a 3-element series capacitor model was used to decouple the “interfacial capacitance” from the “specimen volumetric capacitance” [26,27]. This specimen configuration was adopted herein for the “dielectric configuration”, although the data reduction method used herein measures directly |*Z*| and *θ* without the assumption of a circuit model. The MWCNT/PP specimens were 90 mm long and 12.5 mm wide rectangular strips, with electrodes comprising a dielectric layer directly bonded to the specimen and aluminum foil on top, as advised in [26,27]. Each electrode consisted of a thin (40 µm thick) 12.5 mm side-length square section of aluminum foil and three layers of double-sided adhesive tape (70 µm thick), for a total thickness of 0.25 mm, see Figure 2. Notice that the width of the specimen needed to be larger than the one corresponding to the “resistive configuration” to allow larger electrode area for dielectric measurements. For narrower specimens, the signal to noise ratio was too small in the dielectric configuration.

In the dielectric configuration, impedance (*Z*) measurements were conducted by four-wire measurements placing the *I* and *V* wires at the same location, as is depicted in Figure 2. For frequencies below 1 kHz, the impedance measurements of this configuration exhibited high noise and were not stable. Thus, the frequency response of the specimen in the dielectric configuration was measured from frequencies from 1 kHz to 1 MHz using a 5-test replicate plan. For the electromechanical characterization, a uniaxial tensile load (*P*) was applied until failure to 4 wt.% and 5 wt.% nanocomposites. Strain (*ε*) was calculated from the crosshead displacement of the universal testing machine using a gage length of 50 mm. The piezoimpedance characterization in the dielectric configuration was performed at 100 kHz. Finally, the piezoimpedance sensitivity factors (*k_z_* and *k_θ_*) for the dielectric configuration were determined identically to those of the resistive configuration explained in the previous section using the same strain intervals. The piezoimpedance results are presented and discussed directly from the measured impedance modulus and phase angle of the complex impedance response, rather than from circuit model assumptions.

## 3. Results and Discussion

### 3.1. Frequency Response

**Figure 3:**
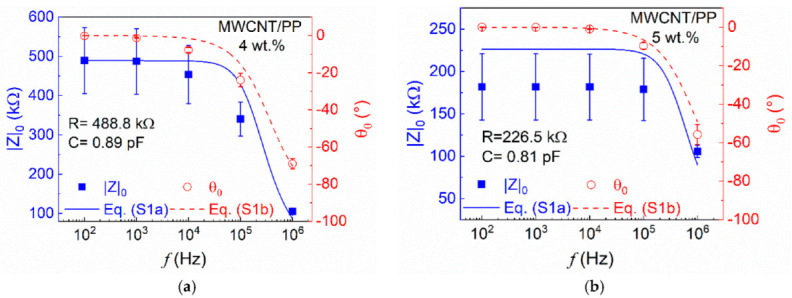
Frequency response of the impedance of MWCNT/PP nanocomposites in the resistive configuration. (**a**) 4 wt.%; (**b**) 5 wt.%.

Figure 3 summarizes the frequency response of five specimens of the MWCNT/PP nanocomposites with 4 wt.% and 5 wt.% in the resistive configuration. Figure 3a shows the (zero-strain) impedance modulus (|*Z*|_0_) and phase angle (*θ*_0_) as a function of frequency (*f*) for 4 wt.% MWCNT/PP nanocomposites. Data points indicate measurements, while the solid and dashed lines represent best fits to Equations (S1a) and (S1b) using the RC parallel circuit model described in Section S.1. For frequencies below 10 kHz, |*Z*|_0_ remains fairly constant (around 490 kΩ, with variations below 0.4%), and *θ*_0_ remains close to zero (~−0.1°). However, for frequencies of 10 kHz and above, *|Z|*_0_ decreases nonlinearly until ~105 kΩ (~80% decrease) at 1 MHz. At the same frequency interval, *θ*_0_ decreases toward more negative values, reaching −70° at 1 MHz. The increase in phase angle means that the electrical current leads the voltage signal. Negative phase angles are indicators of capacitive behavior. This is because capacitors work as electric charge storages; i.e., they cause the voltage to delay with respect to the current [30]. In nanocomposites, this can be explained by considering that, on a micrometric scale, a pair of proximal CNTs separated by a thin layer of insulating polymer can be considered as a micro-capacitor. The CNTs function as electrodes and the polymeric layer between them as the dielectric [3,24,31,32]. Thus, the behavior presented by the impedance (both *|Z|*_0_ and *θ*_0_) indicates a transition from a dominantly resistive response (with negligible capacitive contribution) to an important capacitive contribution for frequencies above 1 kHz. The best fit values of *R* and *C* according to the circuit model of Equations (S1a) and (S1b) are included as insets in Figure 3. The *R* and *C* best fit values listed in Figure 3 also suggest a considerably higher contribution of resistance (~10^5^) over capacitance (~10^−12^) to the total impedance of the system. This is associated with the dielectric properties of the material and the interfacial polarization phenomenon occurring in this kind of nanocomposites [24]. A very similar response is observed for nanocomposites at 5 wt.% in Figure 3b, but the transition where the capacitance contribution becomes relevant is shifted toward higher frequencies (above 10 kHz). Higher MWCNT content in nanocomposites means a more packed conductive network and, hence, less probability for forming micro-capacitors. Since micro-capacitor formation occurs only for noncontacting CNTs, smaller amounts of micro-capacitors are expected for 5 wt.% composites, yielding significant polarization effects only at higher frequencies [31]. In this regard, in microscopically heterogeneous materials such as MWCNT/PP nanocomposites, there is an accumulation of polarized charges at the filler/matrix interfacial region. This is due to the large difference in conductivities and permittivities between the matrix and conductive fillers [32,33]. This interfacial polarization phenomenon is explained by the Maxwell–Wagner–Sillars mechanism [34]. With increasing frequency, there is larger accumulation of charges, the energy in the charge carriers increases, and their passage through the MWCNT–PP interface eases, thus increasing the effective electrical conductivity of the nanocomposite [22]. Thus, the observed behavior indicates that the accumulation of charges at the polymer–nanofiller interface increases nonlinearly with increasing frequency, as has been observed for other similar material systems [24,32,33,35]. However, the interfacial polarization of materials is influenced by factors such as the structure/property relationships of the fillers and the polarizability of the polymer.

In this regard, it was observed that nonpolar polymers such as PP do not yield high interfacial polarization, due to their low dielectric constant (2.2 at 1 MHz) [30]. This explains the low contribution of the capacitive component to *|Z|*_0_ and *θ*_0_ for frequencies below the kHz range. The frequency response in the dielectric configuration is included in Section S.2 of the supplementary information. According to the results discussed herein, frequencies of 10 kHz and 100 kHz were selected for further piezoimpedance characterization.

### 3.2. Piezoimpedance Response

#### 3.2.1. Resistive Configuration

**Figure 4:**
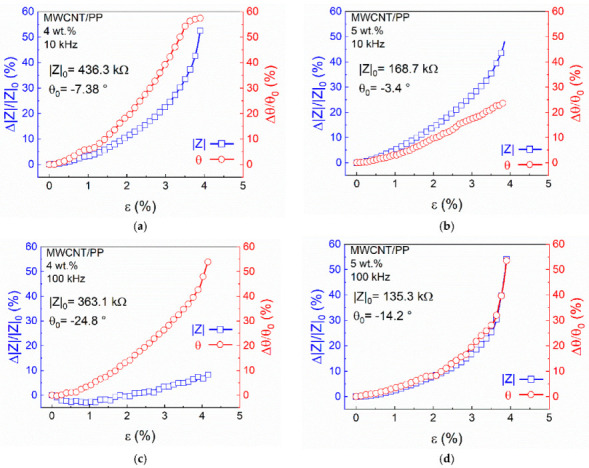
Piezoimpedance response of nanocomposites (resistive configuration) as a function of MWCNT content and frequency. (**a**) 10 kHz (4 wt.%); (**b**) 10 kHz (5 wt.%); (**c**) 100 kHz (4 wt.%); (**d**) 100 kHz (5 wt.%).

Figure 4 shows the representative curves of the piezoimpedance response (*Δ|Z|/|Z|*_0_ and *Δθ/θ*_0_ as a function of ε) of MWCNT/PP composites at *f* = 10 kHz (Figure 4a,b) and *f* = 100 kHz (Figure 4c,d) in the resistive configuration. For all cases, |Z| increases with increased tensile strain, while *θ* increases toward more negative values. Since *θ*_0_ was always (slightly) negative, this renders positive fractional changes *Δ|Z|/|Z|*_0_ and *Δθ/θ*_0_, which increase with the applied strain. For 10 kHz (Figure 4a,b), *Δ|Z|/|Z|*_0_ for both MWCNT concentrations show a nonlinear behavior, with maximum values of *Δ|Z|/|Z|*_0_ ~55% for *ε* = 4%. At the failure strain (*ε* ~4%), *Δθ/θ_0_* ~60% for the 4 wt.% nanocomposites. Lower fractional changes (*Δθ/θ_0_* ~30%) were observed for nanocomposites at 5 wt.%.

In MWCNT/PP nanocomposites, the contribution of the resistive and capacitive components to the total impedance is strongly influenced by the spacing between the conductive elements (CNTs) within the polymer [3,24,36]. In this regard, it is important to point out that it was not attempted to calculate sensitivity (“gage”) factors from the extracted *R* and *C* components of the circuit model. This was deliberately done in order to rely only on measured metrics for the quantification of sensitivity. When nanocomposites are subjected to axial tension, it is expected that the increase in the distance between CNTs will cause the effective value of electrical resistance to increase. On the other hand, the effective capacitance may decrease [15,19] or increase with strain, depending on the relative motion and spacing between conductive fillers. A decrease in capacitance with increased spacing between CNTs can be rationalized by a typical parallel plate capacitor model. In such a model, *C* is inversely proportional to the transverse distance between the conductive elements and directly proportional to the (overlapping) plate area, in a simple one-dimensional description of the problem [30]. Thus, under the assumption of such a simple one-dimensional description, the measured piezoimpedance response (increase of *|Z|* and *θ* with increased strain) indicates that *C* decreases with the applied strain, and that the contribution of the resistive component strongly dominates over the capacitive one. Other factors, such as two-dimensional motions and rotations, may be causing *C* to increase with strain, as will be further discussed in Section 3.3. This response is influenced by the CNT content, as seen in Figure 4b (being more evident for *Δθ/θ*_0_), rendering more sensitivity for nanocomposites at 4 wt.%. As seen from Figure 3b, at 10 kHz, the impedance of 5 wt.% nanocomposites is dominated by the resistance contribution. Indeed, for nanocomposites at 5 wt.%, *Δθ/θ*_0_ reaches maximum values of ~25% at *ε* = 4%, confirming the relatively low contribution of the capacitive component to the impedance of these nanocomposites. The lower piezoimpedance sensitivity observed for nanocomposites with higher CNT content can be explained by the same network saturation arguments as those used for piezoresistivity (DC) in previous works [3,11,12], as outlined in Section S.3 of the supplementary information. For 4 wt.% nanocomposites tested at 100 kHz (Figure 4c), there is an important decrease in the *Δ|Z|/|Z|_0_* sensitivity at the same level of strain than that at 10 kHz. Indeed, all tested replicates showed a slight decrease in |*Z*| for small strains (*ε* < 1%) as seen in Figure 4c. This is explained by the relatively large contribution of capacitance for these nanocomposites (4 wt.% at 100 kHz) and the two-dimensional motion of the CNTs within the polymer during loading. These effects cause *C* to increase with strain, as will be further explained in Section 3.3. The relatively low stiffness and large Poisson ratio of PP compared to other polymers such as epoxy thermosettings, account for increased degrees of freedom of the conductive fillers within the polymer, including rotations and two-dimensional motions. On the other hand, at 100 kHz, the nanocomposites with 5 wt.% (Figure 4d) still exhibit a dominant contribution of the resistance to impedance, showing higher values of *Δ|Z|/|Z|*_0_ than 4 wt.% nanocomposites at the same *f*.

#### 3.2.2. Dielectric Configuration

**Figure 5:**
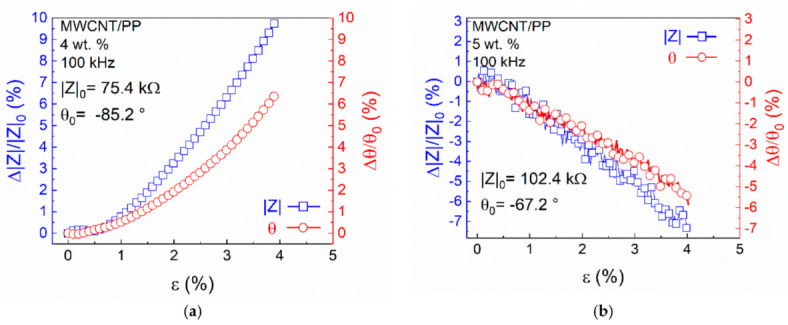
Piezoimpedance response of the nanocomposites in the dielectric configuration. (**a**) 4 wt.%; (**b**) 5 wt.%.

Figure 5 shows the piezoimpedance response of 4 wt.% (Figure 5a) and 5 wt.% (Figure 5b) MWCNT/PP composites tested in the dielectric configuration. In Figure 5a, the curve of *Δ|Z|/|Z|*_0_ vs. *ε* shows that for low strains (*ε* < 0.5%), *Δ|Z|/|Z|*_0_ attains very small values. However, for higher strains, a significant increase in *Δ|Z|/|Z|*_0_ with strain is observed. Similarly, *Δθ/θ*_0_ is small for *ε* < 0.5% and increases nonlinearly thereafter. The maximum values attained (*ε* ≈ 4%) were *Δ|Z|/|Z|*_0_ ≈ 10% and *Δθ/θ*_0_ ≈ 6%. In this configuration, the contribution of the capacitive component to the piezoimpedance response comprises the capacitance of the electrodes and the change in permittivity of the MWCNT/PP composite. However, the capacitance of the electrodes is not expected to significantly change with strain. This can be rationalized considering a parallel plate capacitor, where the capacitance depends on the distance between plates, the overlapping area between plates, and the permittivity of the dielectric between them [30]. For polymeric composites based on carbon nanostructures, the change in the permittivity depends on the change in the dielectric properties of the material, which in turn depend on the interfacial polarization [9,37]. In the dielectric configuration, the response was markedly different for nanocomposites at 5 wt.% (Figure 5b), whose conductivity is rather high. Additional tests of a second batch of other five 5 wt.% MWCNT/PP replicates confirmed this trend. Such nanocomposites exhibited a negative piezoimpedance response for both *|Z|* and *θ*, with maximum changes of *Δ|Z|/|Z|_0_* ≈ −7% and *Δθ/θ_0_* ≈ −5.5%. This correlates well with the frequency response observed in Section S.2 of the supplementary information for 5 wt.% nanocomposites in the dielectric configuration. As seen from Section S.2, the response of *θ*_0_ to frequency variations for 5 wt.% nanocomposites was unexpected and exhibited features of inductive effects, attributed to the loop of the material and experimental setup. Therefore, at this concentration, the contribution of inductive effects seems to become more relevant. In this way, the increment in inductance has an opposite effect to capacitive in the reactance, causing a decrease in *|Z|* and *θ* (toward fewer negative angles) with increased strain.

### 3.3. Comparison of Sensitivity Factors

**Figure 6:**
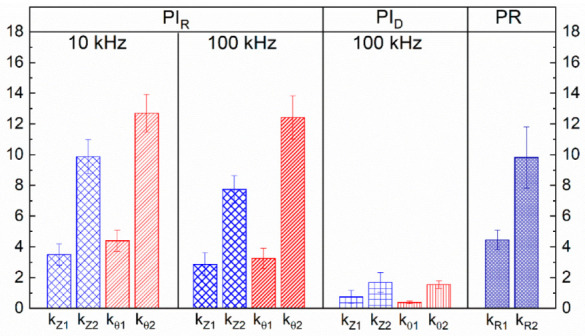
Sensitivity factors for 4 wt.% MWCNT/PP composites in the AC resistive configuration (PI_R_), AC dielectric configuration (PI_C_), and DC (PR).

A summary of the sensitivity factors calculated using Equation (1) from the electromechanical response in AC (resistive and dielectric configuration) and DC of the 4 wt.% MWCNT/PP nanocomposites is presented in Figure 6. In this Figure, the AC resistive configuration is labeled as “*PI_R_*”; the dielectric configuration is labeled as “*PI_C_*”, and the label “*PR*” refers to the electromechanical sensitivity response of nanocomposites in DC (presented in Section S.3 of the supplementary information). Since the mechanical behavior was similar for all specimens (see typical stress–strain curves in Figure S3 of the supplementary information), the sensitivity was calculated at the same strain range for all configurations. The first subscript of the sensitivity factors (*k*) refers to the electrical parameter *(|Z|*, *θ* or *R*), while the second subscript refers to the strain level (“1” for *ε* ≤ 0.8% or “2” for 1% ≤ *ε* ≤ 3%). For all cases, the sensitivity factors (*k_Z_*_1_, *k_θ_*_1,_ and *k_R_*_1_) obtained for the region associated with the elastic regime (*ε* ≤ 0.8%) are lower than those calculated for higher strains (1% ≤ *ε* ≤ 3%). For low strain levels (*ε* ≤ 0.8%), a slightly higher sensitivity was obtained for the *PI_R_* response at 10 kHz than for that at 100 kHz. For both frequencies at *ε* ≤ 0.8%, *k_Z_*_1_ is numerically similar to the values obtained for conventional piezoresistive testing (*k_R_*_1_ = 4.2). The sensitivity of the *PI_C_* configuration was smaller than that of the *PI_R_* and *PR* configurations for any strain level, given the low resistive contribution of this response. For higher strains (1% ≤ *ε* ≤ 3%), a slightly higher sensitivity was obtained for the *PI_R_* response at 10 kHz, with the average *k_Z_*_2_ = 9.9 and *k_θ_*_2_ = 12.7. Those values are slightly higher than the corresponding values at 100 kHz, and *k_θ_*_2_ is significantly higher than the sensitivity obtained in DC (*K_R_*_2_ = 9.8). Thus, while the response of the resistive component demonstrated high sensitivity, increased sensitivity can be obtained by using an AC signal, tuning the frequency used. Increased sensitivity of the *PI_R_* configuration was more obvious from the phase angle (*θ*), with a sensitivity ~30% higher than that of *PR*. Regarding the dielectric configuration (*PI_C_*), the piezoimpedance response turned out to be the least sensitive, with *k_Z_*_1_ = 0.74, *k_θ_*_1_ = 0.38, *k_Z_*_2_ = 1.7, and *k_θ_*_2_ = 1.6. A summary of the sensitivity factors obtained for 5 wt.% nanocomposites is presented in Section S.4 of the supplementary information. Similar trends were observed when comparing the sensitivities obtained by the configuration *PI_R_* to those of *PR*, but such factors were negative for the configuration *PI_C_*. The behavior of *PI_C_* at 5 wt.% is believed to be caused by inductive contributions of the material and setup, as explained in Section S.4 of the supplementary information.

For MWCNT–epoxy composites, other authors have found that the piezoimpedance sensitivity increases with increased frequency [15]. The fact that the sensitivity at 100 kHz is slightly smaller than that at 10 kHz herein suggests that the effect is multifactorial and may also be related to the material system. That is, the relatively low stiffness and high Poisson’s ratio of PP, as well as the properties of the MWCNTs used, may cause the CNT motion inside the polymer to become two- or there-dimensional, including the rotational degrees of freedom. Thus, the relative motion between CNTs governing the piezoimpedance can no longer be thought of as a simple one-dimensional motion (separation or approximation along the loading direction) of the conductive elements. This rationale is multifactorial and requires additional considerations.

**Figure 7:**
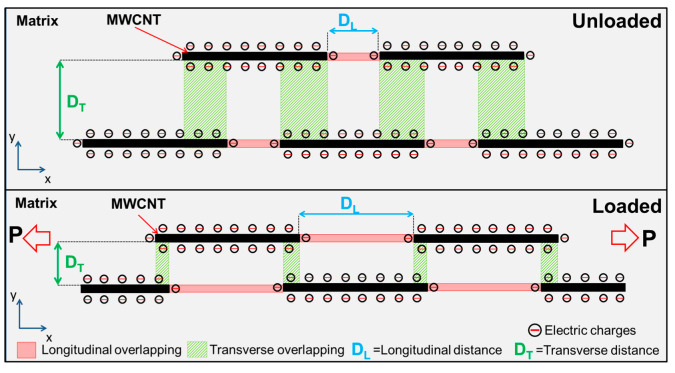
Schematic representation of the CNT–CNT two-dimensional motion inside a flexible polymer upon loading, considering transverse contraction.

In the impedance response, as the frequency increases, the contribution of the capacitive/permittivity effects increases, due to interfacial polarization effects in the nanocomposite [22]. However, the increase in the capacitive/permittivity contribution to the total impedance with increased frequency may not always mean an increase in the piezoimpedance sensitivity, as seen in Figure 6. In nanocomposites such as the MWCNT/PP ones studied here, upon application of uniaxial load/strain, CNTs tend to draw away in the loading direction (*x* direction in Figure 7), increasing the longitudinal distance (*D_L_*) and thus decreasing the capacitance. However, in the transverse (*y*) and through-thickness directions, the CNT to CNT distance (transverse distance, *D_T_*) may decrease due to the transverse (Poisson’s) contraction of the material, as depicted in Figure 7. In this sense, the CNT–CNT system could be rationalized as parallel plate capacitors in the transverse direction, in which the capacitance is directly proportional to the overlapping section and inversely proportional to the distance between the plates [30]. Therefore, the capacitance may increase or decrease upon strain application, depending on the mechanical properties of the polymer matrix. If the effective result of the CNTs motion and rotation upon strain application is an increase in capacitance, both capacitance and resistance increase with strain. According to the parallel model of Equations (S1a) and (S1b), an increase in *C* would yield an increase in *θ* and a decrease in *|Z|.* Thus, if *C* increases with the applied strain, *R* and *C* may yield competing contributions to the piezoimpedance. At low frequencies, the capacitive (permittivity) effects yield a low contribution to the impedance, so the piezoimpedance response is strongly dominated by the resistive contribution in the low frequency regime. However, at high frequencies and large strains, the competing contribution between *R* and *C* with strain may explain the slight decrease in piezoimpedance sensitivity observed for |*Z*| in Figure 6 for *f* = 100 kHz. This effect may become more relevant for a flexible polymer such as PP, whose (measured) elastic modulus is relatively low (1.2 GPa), and whose Poisson’s ratio is high (0.42). It should also be kept in mind that this picture assumes that the permittivity and electrical conductivity of the matrix and filler are constant, and so are the effective *C* and *R*. In practice, for this kind of nanocomposites, the material properties may change with strain, and the effective *C* and *R* used to represent the material as a circuit model could also depend on frequency.

## 4. Conclusions

The electrical and electromechanical (piezoimpedance) responses under alternating current (AC) of MWCNT/PP composites was investigated and compared to their direct current (piezoresistance) counterpart. The frequency (*f*) and piezoimpedance responses were investigated considering two electrode configurations. The first one comprised the conventional copper cables bonded by conductive paint, named “resistive configuration”. The second one was an enhanced-permittivity one, where the capacitive component was expected to be amplified (named “dielectric configuration”). This dielectric configuration was recently suggested by other authors [26] and requires the use of an electrically insulating film between the specimen and the aluminum foil electrodes.

The impedance of MWCNT/PP nanocomposites in the resistive configuration exhibited a resistive–capacitive behavior, which was suitably fitted to an *RC* parallel circuit model. The impedance response presented a strong dominance of the resistive component at low frequencies, with *|Z|*_0_ values that were nearly constant and close to the equivalent *R* values extracted from the *RC* parallel model. The transition to more significant capacitive contributions occurred for frequencies of 10 kHz and higher. The frequency where capacitive contributions started to play a significant role was higher for MWCNT/PP composites at 5 wt.% than for those at 4 wt.%. The capacitive contribution is attributed to the formation of CNT/polymer/CNT micro-capacitors and charge accumulation at the CNT–polymer interface. The frequency response in the dielectric configuration indicated enhancement of the capacitive contribution to the impedance with increased frequency, but also exhibited signs of inductive contributions related to the measurement setup.

The AC electrical response under strain (piezoimpedance) of these nanocomposites was dominated by the resistive component, but it also presented meaningful capacitive contributions when tested at 10 kHz or 100 kHz. The highest sensitivity factors (“gage factors”) were found for the piezoimpedance response of MWCNT/PP composites in the resistive configuration at 4 wt.%. For these materials, the sensitivity factors in the small strain regime (*ε* ≤ 0.8%) was 3.5 for the impedance modulus (*Δ|Z|/|Z|*_0_) and 4.4 for the phase angle (*Δθ*/*θ*_0_). For the large strain regime (1 ≤ *ε* ≤ 3%), the corresponding factors were 9.9 (*Δ|Z|/|Z|*_0_) and 12.7 (*Δθ*/*θ*_0_). These sensitivity factors were higher than their DC (piezoresistive) counterparts. Hence, the AC phase angle arises as a new parameter for quantifying sensitivity for strain sensing applications of smart materials. This parameter would not only render higher sensitivity, but it also provides valuable information on the resistive/capacitive contributions, without the need of a circuit model. The AC concept investigated herein proved to be a viable alternative to increase the electromechanical sensitivity of carbon-nanostructured nanocomposites. Besides generating new knowledge, these findings contribute toward the development of strain and motion sensing devices, particularly those based on flexible polymers, such as tactile sensors for human–machine interfaces as well as soft robotics.

## Data Availability

Supplementary information is published along with this article. Further supporting data is available upon request to the corresponding author.

## Supplementary Materials

The following supporting information can be downloaded at: https://www.mdpi.com/article/10.3390/s22020484/s1, Figure S1. Parallel RC electric circuit model. Figure S2. Frequency response of MWCNT/PP nanocomposites in the dielectric configuration. (a) 4 wt.%, (b) 5 wt.%. Figure S3. Piezoresistive response of MWCNT/PP composites. (a) 4 wt.%, (b) 5 wt.%. Figure S4. SEM images of MWCNT/PP composites. (a) 4 wt.%, (b) 5 wt.%. Figure S5. Sensitivity factors for 5 wt.% MWCNT/PP composites in the AC resistive configuration (PIR), AC dielectric configuration (PIC), and DC (PR).
