# Supplementary material for: A Comparative Study of the Electrical and Electromechanical Responses of Carbon Nanotube/Polypropylene Composites in Alternating and Direct Current
Source: Sensors (Basel). 2022 Jan 9;22(2):484. doi: 10.3390/s22020484 (PMC8781245; doi:10.3390/s22020484)
Supplement: Supplementary file 1 [file sensors-22-00484-s001.zip › sensors-1517143-supplementary.pdf]

A comparative study of the electrical and electromechanical responses of carbon nanotube/polypropylene composites in alternating and direct current

A. Balam[a], R. Pech-Pisté[a], Z. Valdez-Nava[b], F. Gamboa[c], A. Castillo-Atoche[d], F. Avilés[a].

[a] Centro de Investigación Científica de Yucatán A.C., Unidad de Materiales, Calle 43 No.130 entre 32 y 34, Col. Chuburná de Hidalgo. C.P. 97205, Mérida, Yucatán, Mexico.

[b] LAPLACE, Université de Toulouse, CNRS, INPT, UPS, Toulouse, France.

[c] Centro de Investigación y de Estudios Avanzados, Unidad Mérida, Departamento de Física Aplicada, Km. 6 Antigua carretera a Progreso A.P. 73, Cordemex, Mérida, Yucatán, Mexico.

[d] Universidad Autónoma de Yucatán, Facultad de Ingeniería, Av. Industrias no Contaminantes por Periférico Norte A.P. 150, Cordemex, Mérida, Yucatán, Mexico.

Supplementary information

S.1 Equivalent circuit models

According to the frequency response of the resistive configuration discussed in section 3.1 of the main manuscript, the MWCNT/PP nanocomposites exhibit a resistive-capacitive (*RC*) behavior. In order to determine the contributions of the resistance (*R*) and capacitance (*C*) to the total impedance of the nanocomposites, several series and parallel equivalent circuit models were tested. The theoretical impedance response as a function of the frequency (*f*) produced by each model was fitted to the experimental data, to assess the model that better fits the measured frequency response. While a series model could not reproduce the experimental data, the basic RC parallel circuit model (Fig. S1) suffices.

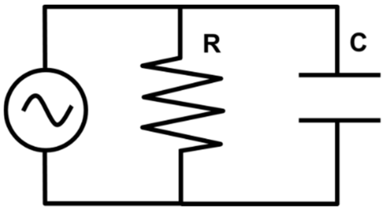

Figure S1 Parallel RC electric circuit model.

The impedance modulus (|*Z*|) and phase angle (θ) of the *RC* parallel circuit model are given by [S1],

$$|Z| = \frac{R}{\sqrt{1+4\pi^2 f^2 R^2 C^2}} \tag{S1a}$$

$$\theta = -\tan^{-1}(2\pi fRC) \tag{S1b}$$

The equivalent circuit model was fitted to experimental data by applying a "global curve fitting" (OriginPro, Northampton, MA, USA) to the $|Z|_0$ and θ_0 frequency responses of nanocomposites in the resistive configuration. The global curve fitting procedure determined the R and C parameters that yield the minimum global error considering both curves, Eq. (S1a) and (S1b). The results of the best fit curves and fitting constants are provided in Fig. 3 of the main manuscript. It is important to mention that adding one or more R, C or RC branches in parallel to the model did not greatly improve the fit to the experimental data.

Regarding the differences between the two MWCNT concentrations, higher values of R (488.8 kΩ) and C (0.89 pF) were found for the nanocomposites with 4 wt.% MWCNT concentration than for those with 5 wt.%. The differences in C between both concentrations are less significant than the differences in R. In this regard, higher concentrations of CNTs promote the formation of a denser network, concomitant with a reduction in the inter-CNT spacing. Less distance between CNTs will decrease the electrical resistance, and might increase the capacitance of the individual CNT-polymer-CNT micro-capacitors, if described as parallel capacitors. However, a high CNT content yields excess of CNT contacts and that may effectively hinder the formation of micro-capacitors [S2]. Therefore, the capacitance may be slightly lower for 5 wt.% nanocomposites than for those at 4 wt.%.

S.2 Frequency response in the dielectric configuration

Figure S2 shows the frequency response of 4 wt.% (Fig. S2a) and 5 wt.% (Fig. S2b) MWCNT/PP composites tested in the dielectric configuration. The lowest frequency at which the impedance of the system could be measured with this configuration was 1 kHz.

For both concentrations, the impedance modulus ($|Z|_0$) decreases logarithmically with the logarithmic increase of f. This tendency indicates an important contribution of the reactance to the total impedance and a low contribution of the resistance, at any frequency tested in this configuration. In the dielectric configuration, since there is no direct contact between the conductive electrodes and the nanocomposite, the resistive contribution to impedance is expected to be minimal. On the contrary, the permittivity/capacitive contribution increase considerably due to the formation of interfacial capacitor systems between the metal

electrodes, the dielectric film and the nanocomposite [S3]. In this regard, the values of θ at 1 kHz indicate a strong capacitive-dominant behavior, since they display angles of ~ -60 ° for 4 wt.% and ~ -90 ° for 5 wt.% nanocomposites. These results confirm that the dielectric configuration enhances the permittivity contribution to total impedance of the system.

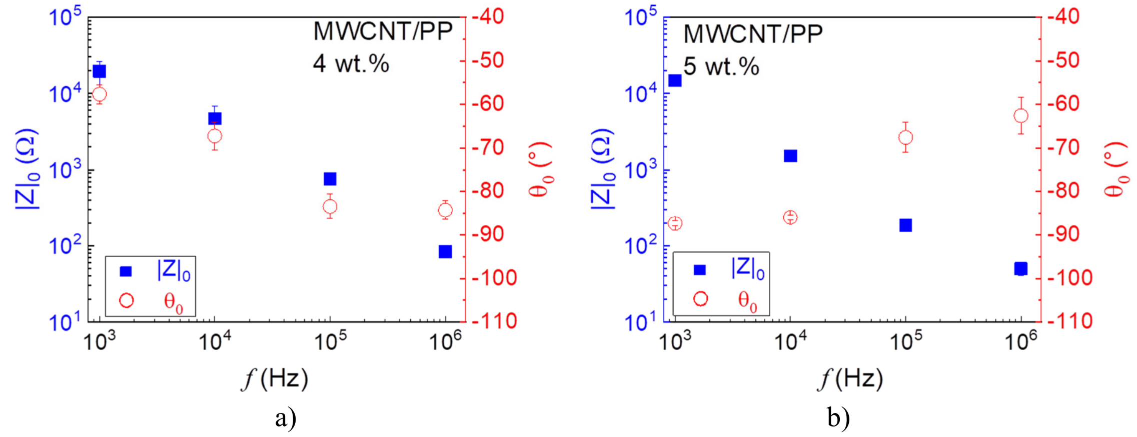

Figure S2 Frequency response of MWCNT/PP nanocomposites in the dielectric configuration. a) 4 wt.%, b) 5 wt.%.

For 4 wt.% nanocomposites θ decreases with increased f, and levels off at 100 kHz. However, for 5 wt.% θ increases towards less negative values with increased f. Ten replicates of this experiment confirmed this unexpected response. Such behavior could be attributed to a parasite inductance of the experimental setup, which becomes more important with increased frequency.

S.3 Piezoresistive response

Figure S3 shows representative curves of the mechanical (stress-strain, σ-ε, left vertical axis) and piezoresistive ($\Delta R/R_0$-ε, right vertical axis) responses of 4 wt.% (Fig. S3a) and 5 wt.% (Fig. S3b) MWCNT/PP composites. Direct current (DC) was used for the results in Fig. S3, i.e. piezoresistivity. For both concentrations, an increase in electrical resistance with increased strain is observed. The piezoresistive response is approximately linear for strains below the yield point of the polymer composite (ε ~ 1.2%), and becomes nonlinear for higher strains. The behavior observed for both concentrations is in agreement with the piezoresistive response of carbon nanostructure-filled PP nanocomposites [S4, S5]. At low strain levels (associated with the elastic region of the nanocomposite), the piezoresistive response is mainly attributed to changes in the morphology of the CNT percolative network, which modifies the spacing, inter-CNT tunneling distance and contact resistance

between CNTs during deformation [S6,S7]. At higher strain levels, the material may experience both reversible and irreversible changes of electrical resistance [S8]. The sensitivity (gage) factors obtained from this test are identified in Fig. 6 and Fig. S.4 with the label "PR", and compared against the results of AC testing (piezoimpedance).

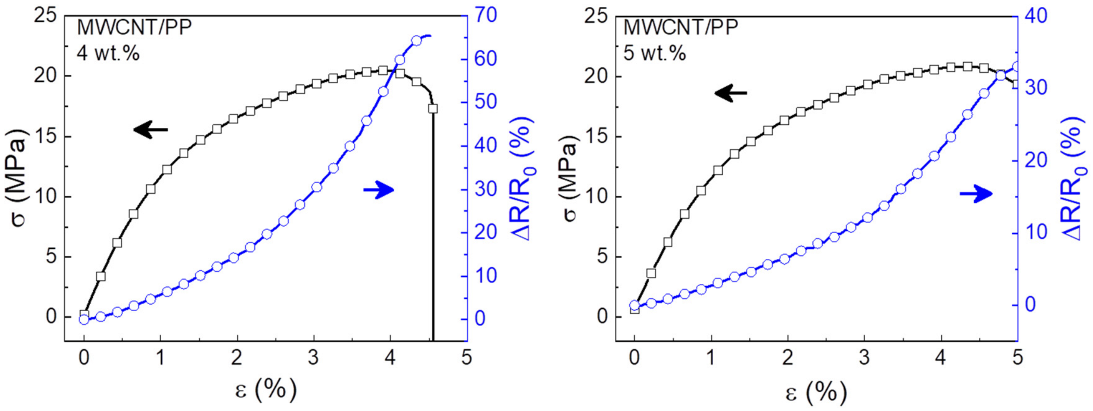

a) b)

Figure S3 Piezoresistive response of MWCNT/PP composites. a) 4 wt.%, b) 5 wt.%.

The reduced sensitivity observed for higher CNT concentrations is due to the denser CNT networks for 5 wt.%. Denser CNT networks for 5 wt.% are observed in the scanning electron microscopies (SEM) of Fig. S4. A denser CNT network yields higher conductivities (~10^{-3} S/cm for 4 wt.% and ~10^{-2} S/cm for 5 wt.%), and experiences less changes in the conductive pathways upon strain application [S6].

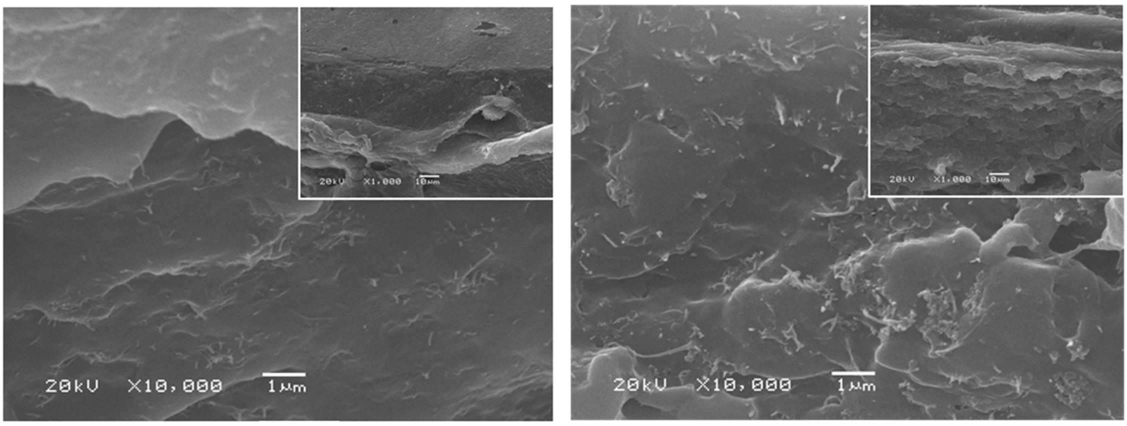

a) b)

Figure S4 SEM images of MWCNT/PP composites. a) 4 wt.%, b) 5 wt.%.

S.4 Comparison of sensitivity factors of nanocomposites with 5 wt.%

Figure S5 summarizes the sensitivity (gage) factors determined for nanocomposites with 5 wt.%. The piezoimpedance response in the resistive configuration (*PI_R*) for both, θ and |Z|, presents higher sensitivity than the piezoresistance response, due to the concurrent contribution of resistance and capacitance. As seen from this figure, nanocomposites with 5 wt.% exhibited lower sensitivity than nanocomposites with 4 wt.% (see Fig. 6). Since both resistive and capacitive contributions depend on the CNT-to-CNT spacing/packing, a denser nanostructured network presumes less electro-sensitivity to strain [S9, S10].

Interestingly, the dielectric configuration (*PI_C*) exhibited negative sensitivity factors for this MWCNT concentration. It has been pointed out (see section 3.2.2, Fig. 5b) that this configuration presents inductive effects possibly due to parasitic induction phenomena yielded by the experimental setup. Such effects may influence the observed decrease in |*Z*| and θ (towards less negative angles) with strain.

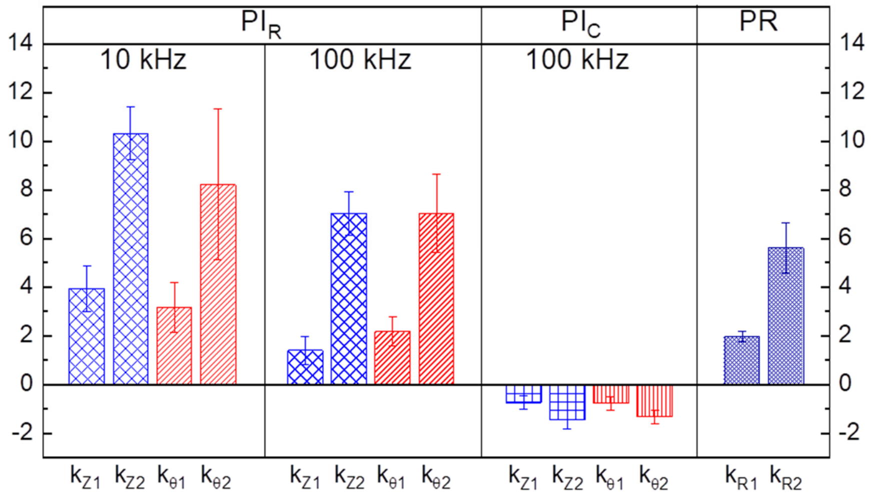

Figure S5 Sensitivity factors for 5 wt.% MWCNT/PP composites in the AC resistive configuration (*PI_R*), AC dielectric configuration (*PI_C*), and DC (*PR*).

References

[S1] J. Bird, Electrical and Electronic Principles and Technology, 6th ed., Routledge, London, UK, (2017). doi:10.4324/9781315561875.

[S2] T.T.N. Dang, S.P. Mahapatra, V. Sridhar, J.K. Kim, K.-J. Kim, H. Kwak, Dielectric properties of nanotube reinforced butyl elastomer composites, J. Appl. Polym. Sci. 113 (2009) 1690–1700. doi:10.1002/app.30166.

[S3] A.A. Eddib, D.D.L. Chung, First report of capacitance-based self-sensing and in-plane electric permittivity of carbon fiber polymer-matrix composite, Carbon 140 (2018) 413–427. doi:10.1016/j.carbon.2018.08.070.

[S4] O. Zetina-Hernández, S. Duarte-Aranda, A. May-Pat, G. Canché-Escamilla, J. Uribe-Calderon, P.I. Gonzalez-Chi, F. Avilés, Coupled electro-mechanical properties of multiwall carbon nanotube/polypropylene composites for strain sensing applications, J. Mater. Sci. 48 (2013) 7587–7593. doi:10.1007/s10853-013-7575-3.

[S5] J. Zhao, K. Dai, C. Liu, G. Zheng, B. Wang, C. Liu, J. Chen, C. Shen, A comparison between strain sensing behaviors of carbon black/polypropylene and carbon nanotubes/polypropylene electrically conductive composites, Compos. Part A Appl. Sci. Manuf. 48 (2013) 129–136. doi:10.1016/j.compositesa.2013.01.004.

[S6] F. Avilés, A.I. Oliva-Avilés, M. Cen-Puc, Piezoresistivity, Strain, and Damage Self-Sensing of Polymer Composites Filled with Carbon Nanostructures, Adv. Eng. Mater. 20 (2018) 1701159. doi:10.1002/adem.201701159.

[S7] D.D.L. Chung, A critical review of piezoresistivity and its application in electrical-resistance-based strain sensing, J. Mater. Sci. 55 (2020) 15367–15396. doi:10.1007/s10853-020-05099-z.

[S8] L.M. Chiacchiarelli, M. Rallini, M. Monti, D. Puglia, J.M. Kenny, L. Torre, The role of irreversible and reversible phenomena in the piezoresistive behavior of graphene epoxy nanocomposites applied to structural health monitoring, Compos. Sci. Technol. 80 (2013) 73–79. doi:10.1016/j.compscitech.2013.03.009.

[S9]A. Sanli, A. Benchirouf, C. Müller, O. Kanoun, Piezoresistive performance characterization of strain sensitive multi-walled carbon nanotube-epoxy nanocomposites, Sensors Actuators A Phys. 254 (2017) 61–68. doi:10.1016/j.sna.2016.12.011.

[S10] L. Vertuccio, L. Guadagno, G. Spinelli, P. Lamberti, V. Tucci, S. Russo, Piezoresistive properties of resin reinforced with carbon nanotubes for health-monitoring of aircraft primary structures, Compos. Part B Eng. 107 (2016) 192–202. doi:10.1016/j.compositesb.2016.09.061.
